# Nastiness

**Published:** 1899-12

**Authors:** Chas. B. Gilbert

**Affiliations:** Washington, D. C.


### NASTINESS.

CHAS. B. GILBERT, M. D., WASHINGTON, D. C.

On October 17th, 1893, I was called to see baby W., six months old. I found a miniature old man, dried up to a skeleton; he had never smiled, but whined and cried most of the time; his stools were white, stinking, and turned green on standing in the diaper; vomited most of his milk, being bottlefed. Made no change in milk; gave Psorinum (Hering), 42 m. (Fincke), one dose; on the 21st he looked a little better; on the 27th he was much better, the stools having changed a little to yellow, and he cried less; on the 29th he had a severe vomiting spell after fretting for some time, followed by exhaustion, but after recovering somewhat, he looked up into his mother's face and smiled for the first time.

On December 25th, having gained much flesh, he began to be colicky and to cry much at night, but under proper treatment soon got better; to-day, March 30, 1894, he is so fat that his wrists show the creases and he is a picture of health.

Go to! thou immaculate, who holdest up thy hands at Psor. 42 M. and then injecteth an astringent into a gonorrhœic urethea to stop the flowing out.

